# 5-Hydroxymethylcytosine and ten-eleven translocation dioxygenases in head and neck carcinoma

**DOI:** 10.7150/jca.34806

**Published:** 2019-08-28

**Authors:** Kiyoshi Misawa, Satoshi Yamada, Masato Mima, Takuya Nakagawa, Tomoya Kurokawa, Atsushi Imai, Daiki Mochizuki, Kotaro Morita, Ryuji Ishikawa, Shiori Endo, Yuki Misawa

**Affiliations:** 1 Department of Otolaryngology/Head and Neck Surgery, Hamamatsu University School of Medicine, Hamamatsu, Japan; 2 Department of Otorhinolaryngology/Head and Neck Surgery, Graduate School of Medicine, Chiba University, Chiba, Japan

**Keywords:** 5-hmC, ELISA, TET, HNSCC, disease-free survival

## Abstract

Ten-eleven translocation (TET) enzymes are implicated in DNA demethylation through dioxygenase activity, which converts 5-methylcytosine to 5-hydroxymethylcytosine (5-hmC). However, the specific roles of TET enzymes and 5-hmC levels in head and neck squamous cell carcinoma (HNSCC) have not yet been evaluated. In this study, we analyzed 5-hmC levels and *TET* mRNA expression in a well-characterized dataset of 117 matched pairs of HNSCC tissues and normal tissues. 5-hmC levels and *TET* mRNA expression were examined via enzyme-linked immunosorbent assay and quantitative real-time PCR, respectively. 5-hmC levels were evaluated according to various clinical characteristics and prognostic implications. Notably, we found that 5-hmC levels were significantly correlated with tumor stage (*P* = 0.032) and recurrence (*P* = 0.018). Univariate analysis revealed that low levels of 5-hmC were correlated with poor disease-free survival (DFS; log-rank test, *P* = 0.038). The expression of *TET* family genes was not associated with outcomes. In multivariate analysis, low levels of 5-hmC were evaluated as a significant independent prognostic factor of DFS (hazard ratio: 2.352, 95% confidence interval: 1.136-4.896; *P* = 0.021). Taken together, our findings showed that reduction of *TET* family gene expression and subsequent low levels of 5-hmC may affect the development of HNSCC.

## Introduction

Head and neck squamous cell carcinomas (HNSCCs) are heterogeneous diseases that involve multiple sites and cellular origins within the upper aerodigestive tract 1. Despite aggressive multimodal treatment, survival for patients with HNSCC remains poor. Nevertheless, some patients survive much longer than expected 2. Therefore, identification of prognostic biomarkers as clinical or biological characteristics that provide information on the likely health outcomes of patients, irrespective of the treatment, is essential 3, 4. In HNSCC, epigenetic inactivation associated with tumor-suppressor genes (TSGs) is more frequent than somatic mutations and may drive tumorigenic and progression potential 5-7. Aberrant gene promoter methylation is a key event in cancer development and has attracted increasing interest in basic and translational oncology studies because of the induction of reversible chemical modifications 8, 9.

Enzymes of the ten-eleven translocation (TET) family catalyze the stepwise oxidation of 5-methylcytosine (5-mC) in DNA to 5-hydroxymethylcytosine (5-hmC) and further oxidation products, not only generating new epigenetic marks but also initiating active or passive demethylation pathways 10. Although tissue- and cell type-specific variations occur, it has been estimated that approximately 5% of all cytosines in the genome of mammalian cells are marked as 5-mC, and less than 1% are marked as 5-hmC. Moreover, 5-formylcytosine (5-fC) and 5-carboxylcytosine (5-caC) are 10-1000-fold less abundant than 5-hmC 11, 12. Accordingly, 5-fC and 5-caC may simply be short-lived intermediates in the active demethylation process, whereas 5-hmC may be an active epigenetic mark that is stably maintained 13. TET family proteins can convert 5-mC to 5-hmC, which is widely accepted as the sixth base in the mammalian genome, following 5-mC, the fifth base 14, 15. The few clinical investigations that examined global DNA hydroxymethylation in relation to HNSCC have used genomic DNA from tumors.

Missense and truncating mutations in *TET* genes are present in nearly all solid tumor types at a relatively low frequency 16. In the Cancer Genome Atlas cohort of HNSCC, *TET1*, *TET2*, and *TET3* mutations were identified in nine (1.8%), eight patients (1.6%), and eight patients (1.6%) of 510 patients, respectively 17. Our report indicated that *TET* mRNA is downregulated in HNSCC owing to DNA methylation; this may be a critical event in HNSCC progression. In particular, *TET3* methylation confers HNSCC with unique clinicopathological features 18.

Recent studies have shown that aberrant levels of *TET* genes and 5-hmC are associated with tumorigenesis in different types of cancers 19. In a number of cancers, 5-hmC has been shown to be markedly decreased and associated with tumorigenesis, progression, and outcomes 20. Simultaneous analyses of 5-hmC and *TET* genes are important for predicting tumorigenesis and biological behaviors and for the development of future targeted therapies for HNSCC. However, systematic studies of the epigenetic and transcriptional regulation of 5-hmC and *TET* genes in HNSCC are still needed.

Accordingly, in this study, we compared the 5-hmC profiles between normal mucosa and HNSCC tissues and characterized the associations between 5-hmC and HNSCC tumorigenesis, progression, and outcomes.

## Methods

### Tumor samples

In total, 117 primary HNSCC samples were obtained from patients during surgery at the Department of Otolaryngology, Hamamatsu University School of Medicine. All patients provided written informed consent, and the study protocol was approved by the Institutional Review Board of the Hamamatsu University School of Medicine (date of board approval: 2 October 2015, ethic code: 25-149). Clinical information, including age, sex, alcohol exposure, smoking status, tumor size, human papilloma virus (HPV) status, tumor size, lymph node status, stage, and recurrence, were obtained from the patients' clinical records.

### DNA extraction and ELISA for 5-hmC quantification

The genomic DNA from 117 primary tumors and noncancerous mucosa was extracted using a QIAamp DNA Mini Kit (Qiagen, Hilden, Germany) according to the manufacturer's instructions. The 5hmC content of genomic DNA was determined with a Quest 5-hmC DNA ELISA Kit (Zymo Research, Irvine, CA, USA), according to the manufacturer's instructions. Assays were performed using 4 μg/mL anti-5-hmC polyclonal antibodies, loading 200 ng of DNA per well. Absorbance at 430 nm was evaluated using a SynergyH1 microplate reader and Gen5 software (BioTek, Winooski, VT, USA). The amount of 5-hmC was calculated as a percentage based on a standard curve generated using kit controls.

### RNA extraction and qRT-PCR

Total RNA was isolated using an RNeasy Plus Mini Kit (Qiagen), and cDNA was synthesized using a ReverTra Ace qPCR RT Kit (Toyobo, Tokyo, Japan). The mRNA levels of *TET1*, *TET2*, *TET3*, and glyceraldehyde 3-phosphate dehydrogenase (*GAPDH*) were measured via qRT-PCR using SYBR Premix Ex Taq (Takara, Tokyo, Japan) and a Takara Thermal Cycler Dice Real Time System TP8000 (Takara). The data were analyzed using the ΔΔCt method. Primer sequences were as follows: *TET1* forward (F), CCCTTGGAAATGCCATAGGAA; *TET1* reverse (R), GAGAGCCTGCTGGAACTGTTG; *TET2* F, GGCTGTTGGCCAGAGACTTA; *TET2* R, ATACCTGTAGGTGTTTGCCTGTTTA; *TET3* F, GCCAACTTCAACATACCCTGGAC; *TET3* R, CACCTGGATGTGGGACTGTGTAA; *GAPDH* F, GCACCGTCAAGGCTGAGAAC; *GAPDH* R, TGGTGAAGACGCCAGTCTCTA.

### Data analysis and Statistics

The 5-hmC and TET mRNA levels in tumors and normal mucosa and patient characteristics were analyzed statistically. Receiver-operator characteristic (ROC) curve analyses were performed for 5-hmC and *TET* mRNA levels and all patients for comparisons between tumor and normal tissues. DFS was measured from the date of the initial treatment to the date of diagnosis. Kaplan-Meier tests were used to calculate survival probabilities, and log-rank tests were used to compare survival rates. The prognostic value of methylation status was assessed by performing multivariate Cox proportional hazards analysis adjusting for age (≥ 65 versus < 65 years), sex, smoking status, alcohol intake, and tumor stage (I, II, and III versus IV). A p-value less than 0.05 was considered statistically significant. Statistical analyses were performed using StatMate IV software (ATMS Co. Ltd., Tokyo, Japan) and the Stata/SE 13.0 system (Stata Corporation, TX, USA).

## Results

### Determination of 5-hmC levels by ELISA in HNSCCs and matched normal mucosa

**Figure 1:**
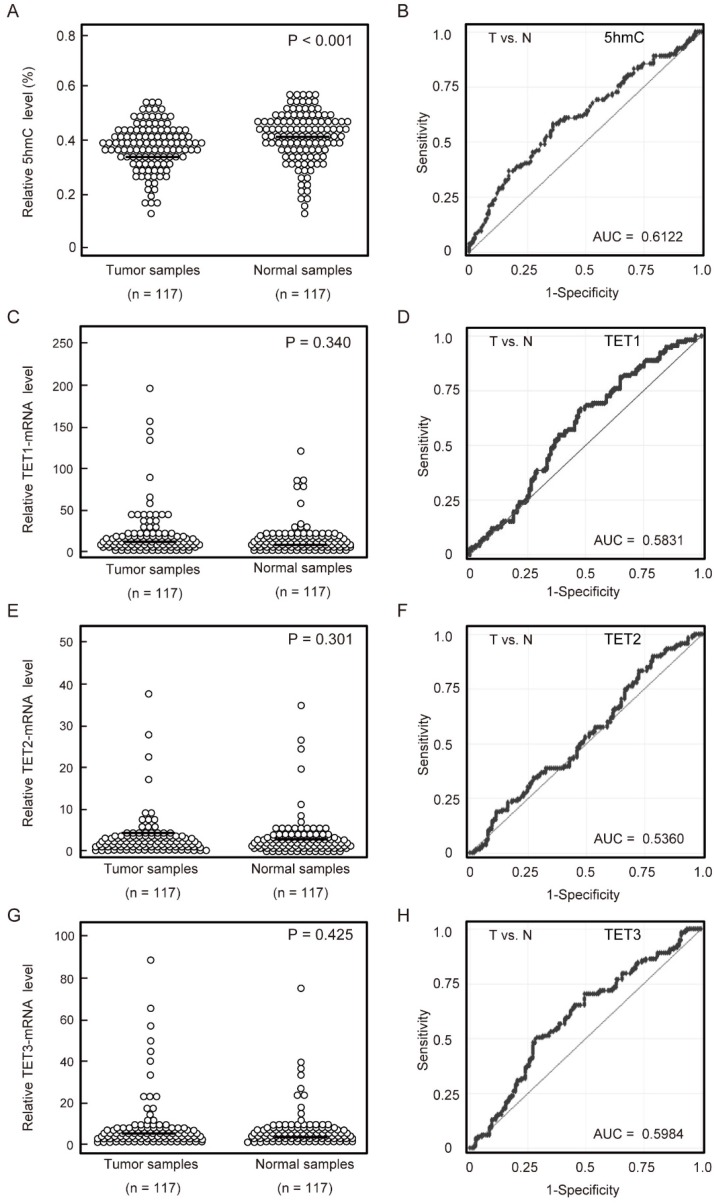
** 5-hmC levels and TET mRNA patterns in matched pairs of HNSCC tissues and adjacent normal mucosal tissues.** (A) ELISAs were used to determine the percentages of 5-hmC levels in 117 matched pairs of HNSCC and normal mucosa specimens (P < 0.001). (B) The AUROC value for 5-hmC was 0.6122. At the cutoff value of 0.407, the sensitivity was 57.3%, and the specificity was 64.1%. (C) Relative TET1 mRNA expression levels (P = 0.340). (D) The AUROC value for TET1 was 0.583. At the cutoff value of 0.580, the sensitivity was 66.7%, and the specificity was 52.1%. (E) Relative TET2 mRNA expression levels (P = 0.301). (F) The AUROC value for TET2 was 0.536. At the cutoff value of 0.1015, the sensitivity was 82.9%, and the specificity was 27.4%. (G) Relative TET3 mRNA expression levels (P = 0.425). (H) The AUROC value for TET3 was 0.598. At the cutoff value of 1.866, the sensitivity was 50.4%, and the specificity was 70.9%. The significance of differences between cancerous and normal mucosal tissues were determined by Student's t‑tests. **P < 0.001.

First, we examined the 5-hmC contents of DNA in 117 matched pairs of HNSCC and matched normal mucosa using ELISA. Cancer tissues had significantly lower levels of 5-hmC (0.373% ± 0.087%) than matched normal mucosa (0.406% ± 0.090%; *P* < 0.001 by paired t-tests). Notably, the 5-hmC levels exhibited highly discriminative ROC curve profiles, which clearly distinguished HNSCC from normal mucosal tissues (area under the ROC [AUROC] = 0.612). At the cutoff value of 0.407, the sensitivity and specificity were 57.3% and 64.1%, respectively (Figure 1A, 1B).

### TET expression in HNSCCs and matched normal mucosa

Quantitative reverse transcription polymerase chain reaction (qRT-PCR) was performed to examine mRNA expression of *TET1*, *TET2*, and *TET3* in 117 matched pairs of HNSCC and normal mucosa. There were no significant differences in *TET1*, *TET2*, and *TET3* mRNA levels between cancerous and normal tissues (Figure 1C, 1E, 1G). *TET1*, *TET2*, and *TET3* mRNA levels showed discriminative ROC curve profiles, which distinguished HNSCC from normal mucosal tissues (AUROC = 0.583, 0.536, and 0.598, respectively). Tumor samples were classified as positive when the mRNA expression levels exceeded 0.580, 0.102, and 1.866 for *TET1*, *TET2*, and* TET3*, respectively. The cutoff mRNA expression level was chosen from the ROC curve to maximize sensitivity and specificity (Figure 1D, 1F, 1H).

### Comparison of 5-hmC levels and TET expression in HNSCC tissues

**Figure 2:**
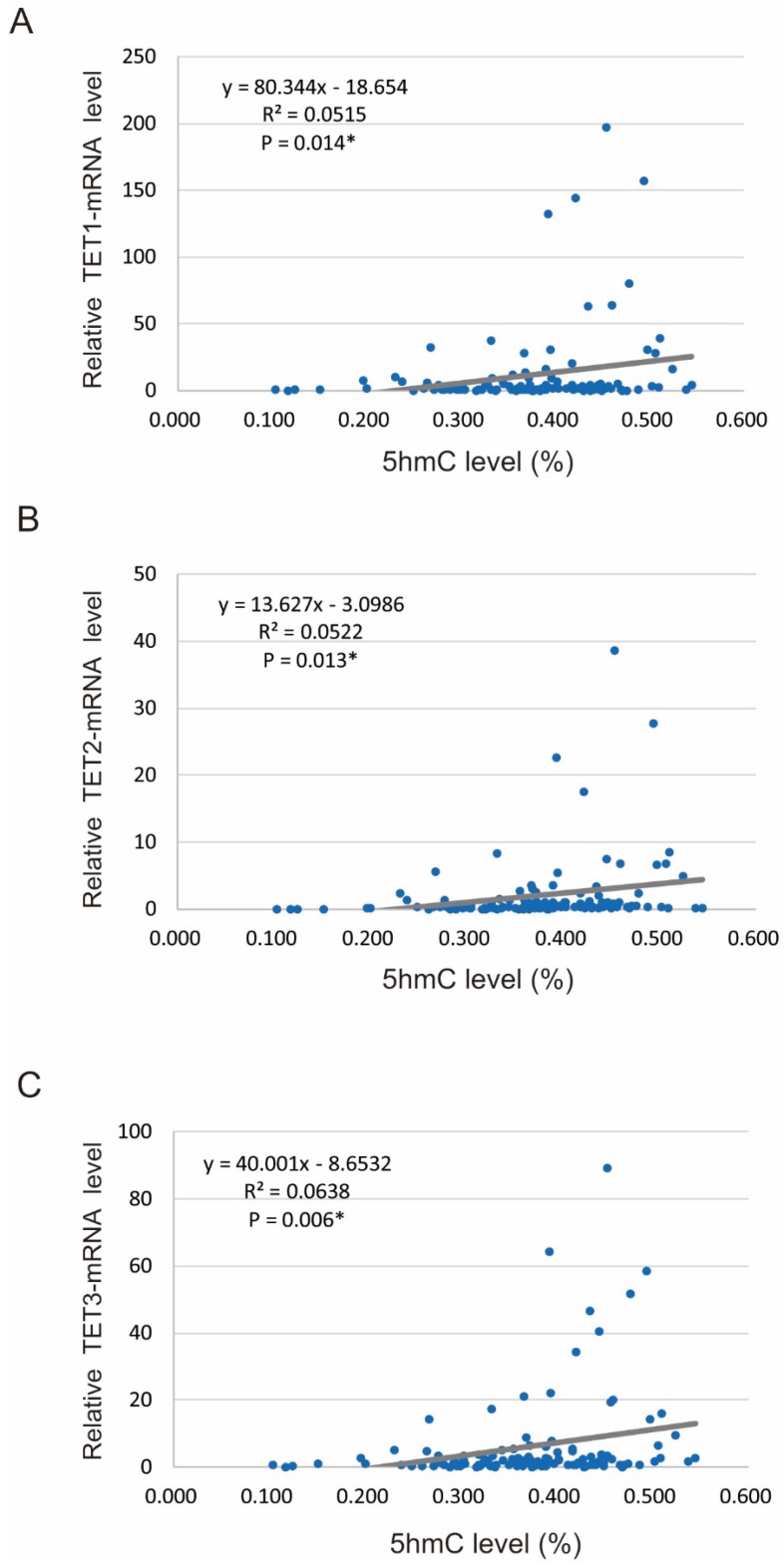
** Spearman rank correlations between 5-hmC and TET mRNA levels in 117 HNSCCs.** (A) Correlation between 5-hmC levels and TET1 expression (R^2^ = 0.052, P = 0.014). (B) Correlation between 5-hmC levels and TET2 expression (R^2^ = 0.052, P = 0.013). (C) Correlation between 5-hmC levels and TET3 expression (R^2^ = 0.064, P = 0.006).

We found that 5-hmC levels were significantly correlated with the relative mRNA levels of *TET* genes, including *TET1* (R^2^ = 0.0515, *P* = 0.014), *TET2* (R^2^ = 0.052, *P* = 0.013), and *TET3* (R^2^ = 0.064, *P* = 0.006) (Figure 2A-C).

**Figure 3:**
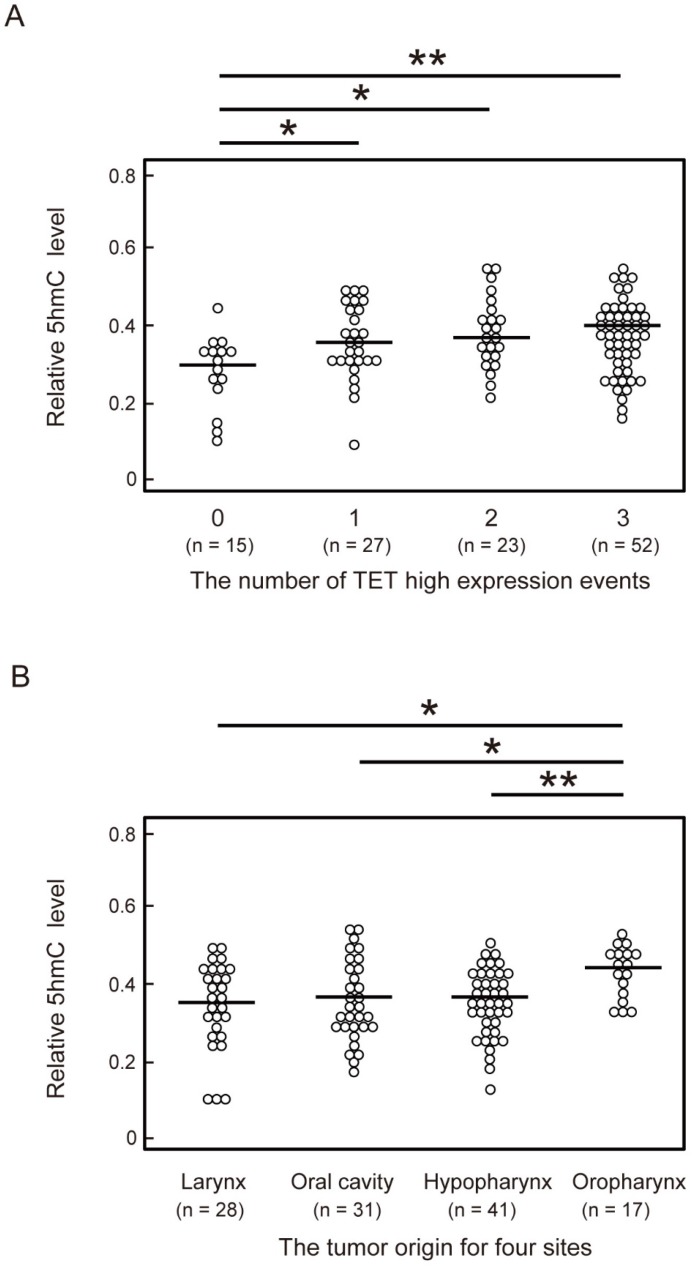
** Comparison of 5-hmC levels and the number of TET high-expression events or the anatomical location of 117 HNSCCs.** (A) Relationship between number of TET high-expression events and 5-hmC levels. 0: all TET genes low expression; 1: one TET genes high expression; 2: two TET genes high expression; 3: all TET genes high expression. (B) Relationship between the anatomical location of the tumor and 5-hmC levels. The significance of relationships between 5-hmC levels and other factors was compared using Student's t-tests. *P < 0.05; **P < 0.01; ***P < 0.001.

To identify factors affecting 5-hmC levels in HNSCC, we compared 5-hmC levels among the number of highly expressed TET genes and tumor sites of HNSCC. One or more *TET* high-expression events were associated with a significant increase in 5-hmC levels compared with no *TET* high-expression events (*P* < 0.05). The 5-hmC level showed the greatest increase when all three* TET* genes showed high expression (*P* < 0.001; Figure 3A). Moreover, in a comparison of 5-hmC levels at tumor sites among HNSCC, 5-hmC levels were found to be significantly higher in patients with oropharyngeal cancer than in patients with larynx (*P* = 0.019), oral cavity (*P* = 0.029), and hypopharynx (*P* = 0.009) cancer (Figure 3B).

### Association of 5-hmC levels and TET expression with clinicopathological assessment

Among the 117 patients, differences in 5-hmC levels and *TET1*, *TET2*, and *TET3* mRNA expression statuses according to clinical information were examined using Chi-squared tests (Table 1). The characteristics of patients with HNSCC are shown in Table S1. We found that 5-hmC levels were associated with clinical stage (*P* = 0.032) and recurrence (*P* = 0.018). Other clinical information, including age, sex, alcohol exposure, smoking status, tumor size, HPV status, tumor size, and lymph node status, was not related to 5-hmC levels. Smoking habit was associated with mRNA expression of *TET1* (*P* = 0.031) and *TET2* (*P* = 0.040). Other clinical information was not related to *TET1*, *TET2*, and *TET3* mRNA expression (Table 1). Comparison of *TET1*, *TET2*, and *TET3* mRNA expression in patients with laryngeal cancer, oral cancer, hypopharyngeal cancer, and oropharyngeal cancer are shown in Figure S1.

### 5-hmC levels and TET expression in HNSCC and the relationship with patient survival

**Figure 4:**
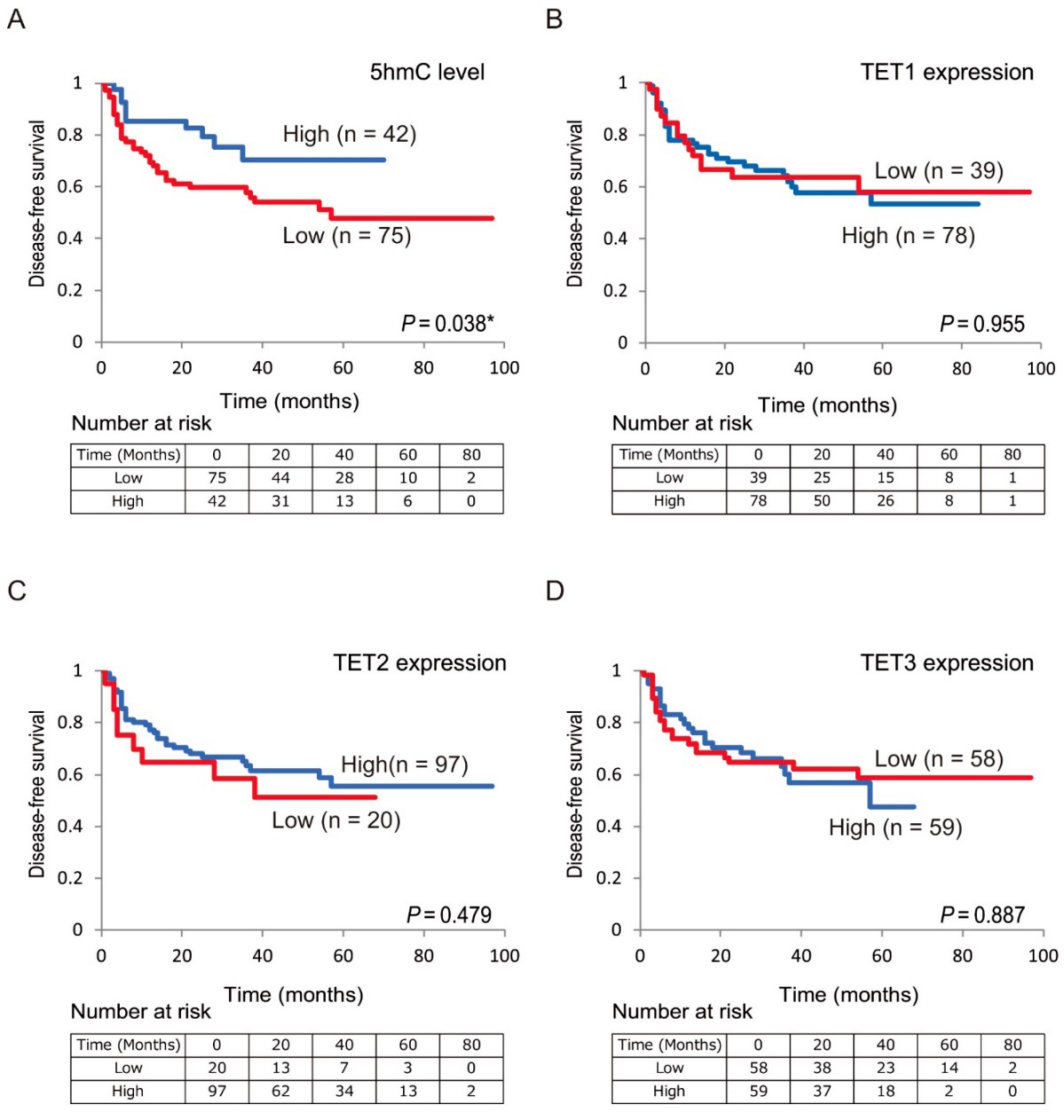
** Kaplan-Meier survival curves based on 5-hmC levels and TET expression status in patients with HNSCC.** DFS according to (A) 5-hmC levels; (B) TET1 expression status; (C) TET2 expression status; (D) TET3 expression status.

Next, we confirmed the relationship between DFS in patients with HNSCC and 5-hmC levels/*TET* expression using Kaplan-Meier plots (Figure 4). Shorter DFS times were observed in patients with low 5-hmC levels compared with those with high 5-hmC levels (log-rank test, *P* = 0.038; Figure 4A). There were no relationships in DFS between the high and low expression groups for *TET1* (78 versus 39, *P* = 0.955), *TET2* (97 versus 20, *P* = 0.479), and *TET3* (59 versus 58, *P* = 0.887) among the 117 patients enrolled in this study (Figure 4B-D).

**Figure 5:**
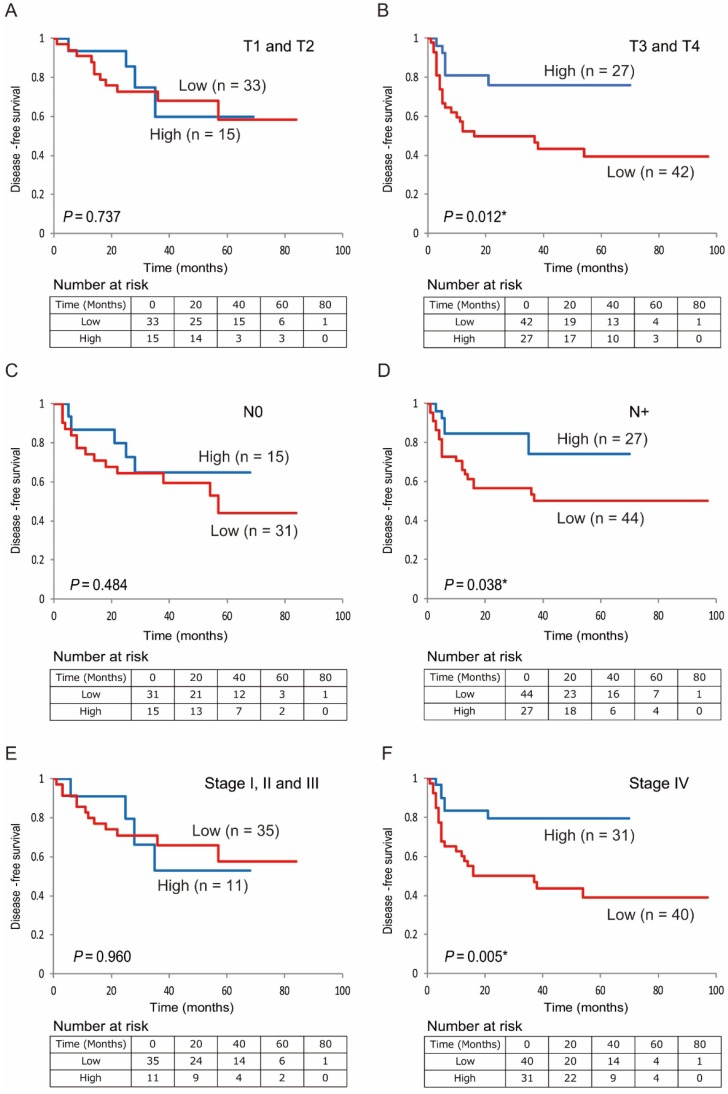
** Kaplan-Meier survival curves based on 5-hmC levels in patients with HNSCC.** DFS for (A) tumor size in T1 and T2 cases (n = 48); (B) tumor size in T3 and T4 cases (n = 69); (C) lymph node status in N0 cases (n = 46); (D) lymph node status in N+ cases (n = 71); (E) stage I, II, and III cases (n = 46); and (F) stage IV cases (n = 71).

Additionally, low 5-hmC levels were associated with decreased DFS compared with high 5-hmC levels in patients with T3 and T4 stages, positive lymph node metastasis, and stage IV disease (*P* < 0.001, *P* = 0.029, *P* < 0.001, and *P* < 0.001, respectively; Figure 5B, 5D, 5F). 5-hmC levels in patients with T1 and T2 stages; negative lymph node metastasis; and stages I, II, and III tumors were not related to outcomes (Figure 5A, 5C, 5E).

**Figure 6:**
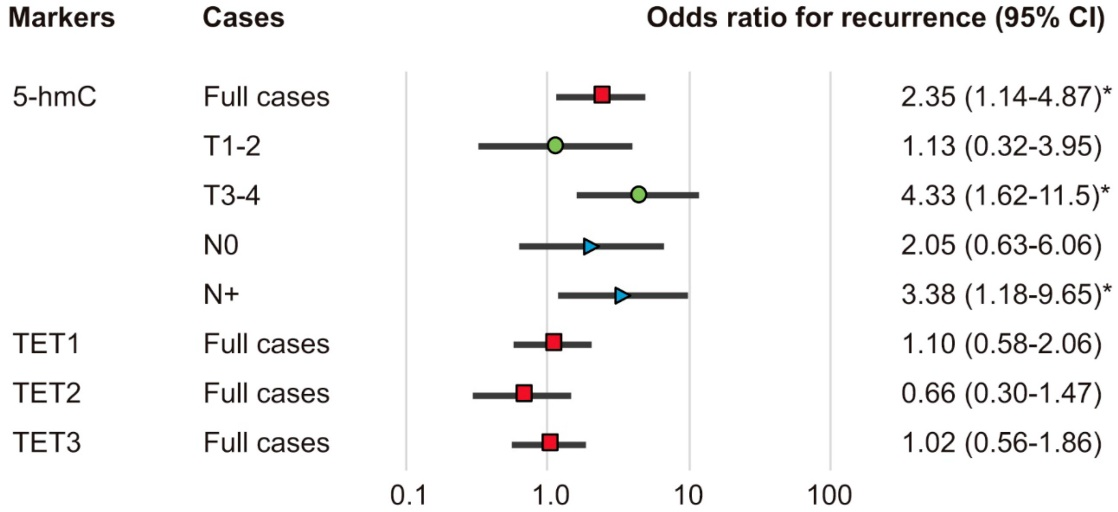
** Odds ratios for recurrence based on Cox proportional hazards models.** Cox proportional hazards model, revealing the estimated odds of recurrence associated with 5-hmC levels and TET1, TET2, and TET3 expression; CI: confidence interval. *P < 0.05.

The associations of the risk of recurrence with 5-hmC levels and *TET1*, *TET2*, and *TET3* statuses were estimated using multivariate analysis with Cox proportional hazards models adjusted for age, sex, smoking status, alcohol exposure, and stage. In patients with high 5-hmC levels (64.1%), the adjusted risk ratio for recurrence (RR) was 2.352 (95% confidence interval [CI]: 1.136-4.869, *P* = 0.021). 5-hmC levels correlated positively with recurrence in patients with T3 and T4 tumor stages and positive lymph node metastasis (RR, 4.33; 95% CI, 1.62-11.5; *P* = 0.003 and RR, 3.38; 95% CI, 1.18-9.65; *P* = 0.023, respectively; Figure 6).

## Discussion

This is the first study examining 5-hmC and *TET* family gene levels in HNSCC. DNA methylation regulates epigenetic gene inactivation; however, the factors affecting DNA demethylation are still poorly understood in HNSCC. Recently, we showed that concurrent methylation analysis of *TET* genes was related to reduced DFS in unfavorable event groups 18. Our current study found that aberrant expression of *TET* genes and altered levels of 5-hmC were associated with tumorigenesis and that lower 5-hmC levels were correlated with reduced survival.

Loss of 5-hmC is associated with decreased expression of TET1 and TET2 in small intestinal neuroendocrine tumors 21. Moreover, 5-hmC levels are significantly reduced in prostate cancer compared with normal prostate tissue samples 22. In esophageal cancer tissues, 5-hmC expression is associated with shorter overall survival and TET2 expression levels 23. TET proteins catalyze DNA CpG demethylation through converting 5-mC to 5-hmC, maintaining a delicate balance between CpG methylation and demethylation in normal cells 24. Notably, promoter CpG methylation-mediated silencing of the* TET1* gene further increases 5-mC levels in tumor cells, thus forming a DNA methylation feedback loop mediated by DNMT and TET1 25.

5-hmC is not simply an activating epigenetic mark, but is considered an intermediate in the active demethylation pathway and appears to play complex roles in gene regulation 11, 12. 5-hmC levels of protein-coding genes are positively correlated with RNA expression intensity 26. A pathway recently suggested for active DNA demethylation in the early mouse embryo involves the conversion of 5-mC to 5-hmC mediated by TET3, which is expressed at high levels in oocytes and zygotes 27, 28. Future studies are needed to confirm the associations between 5-hmC and carcinogenesis and to examine potential mechanisms through which 5-hmC loss affects tumor growth.

Bisulfite treatment, the gold-standard technology for detection of DNA methylation, results in the conversion of unmethylated cytosine into uracil, which will be read as thymine after PCR amplification, with both 5-mC and 5-hmC being read as cytosine, cannot distinguish between 5-mC and 5-hmC 29. Therefore, quantitative analysis of genome-wide distribution of these epigenetic marks has been considered for clinical applications 30. Immuno-based assays, including dot blots, immunohistochemical assays, and ELISA, have widely been used as a quantitative method due to their analytical merits for analyses of 5-hmC 31. Several approaches for 5-hmC mapping have been developed in recent years. Cell-free 5-hmC may represent a new approach for liquid biopsy-based diagnosis and prognosis 32, 33. The 5-hmC profiles of cell-free DNA have been detected in patients with cancer, and 5-hmC gains in both gene bodies and promoter regions have been evaluated in patients with cancer and healthy controls 34. Further studies of the loss of 5-hmC upon transformation of tissues may offer useful tools for dissecting 5-hmC biology in cancers.

In summary, we demonstrated for the first time that 5-hmC levels were abnormally reduced in patients with HNSCC; this may be a critical event in HNSCC progression. Interestingly, the 5-hmC profiles in primary tumors may be used to identify patients with positive lymph node metastasis and high tumor stage that are at a higher risk of recurrence. Further studies are needed to examine the differences in global demethylation patterns observed between 5-hmC-low and -high tumors and their effects on the onset and progression of HNSCC in more detail.

## Supplementary Material

Supplementary figure and table.

## Figures and Tables

**Table 1:** Distribution of 5hmC level and TETs expression status by selected epidemiologic and clinical characteristics

Characteristics			Age			Gender			HPV status		
Markers	Status	Overall (%)	< 65	> 65	P ^†^	Female	Male	P ^†^	positive	negative	P ^†^
5hmC level	High	75 (64.1%)	20	22		6	36		8	34	
	Low	42 (35.9%)	22	53	1	8	67	1	6	69	0.135
TET1 expression	High	78 (66.7%)	27	51		11	67		11	67	
	Low	39 (33.3%)	15	24	1	3	36	0.38	3	36	0.38
TET2 expression	High	97 (82.9%)	34	63		14	83		13	84	
	Low	20 (17.1%)	8	12	1	0	20	0.124	1	19	0.459
TET3 expression	High	59 (50.4%)	22	37		7	52		7	52	
	Low	58 (49.6%)	20	38	0.848	7	51	1	7	51	0.572
Characteristics		Alcohol exposure			Smoking status			Tumor size			
Markers	Status	drinker	non drinker	P ^†^	smoker	non smoker	P ^†^	T1-2	T3-4	P ^†^	
5hmC level	High	36	6		36	6		15	27		
	Low	58	17	0.337	62	13	0.796	33	42	0.436	
TET1 expression	High	61	17		61	17		33	45		
	Low	33	6	0.469	37	2	0.031*	15	24	0.842	
TET2 expression	High	78	19		78	19		39	58		
	Low	16	4	1	20	0	0.040*	9	11	1	
TET3 expression	High	49	10		47	12		27	32		
	Low	45	13	0.493	51	7	0.317	21	37	0.349	
Characteristics		Lympho-node status			Stage			Recurrence events			
Markers	Status	N0	N+	P ^†^	I, II, III	IV	P †	positive	negative	P ^†^	
5hmC level	High	15	27		11	31		10	32		
	Low	31	44	0.693	35	40	0.032*	35	40	0.018*	
TET1 expression	High	29	49		31	47		30	48		
	Low	17	22	1	15	24	1	15	24	1	
TET2 expression	High	35	62		36	61		36	61		
	Low	11	9	0.136	10	10	1	9	11	1	
TET3 expression	High	22	37		25	34		23	36		
	Low	24	34	0.707	21	37	0.571	22	36	1	

† Chi-squared test * P<0.05
