# Supplementary material for: 5-Hydroxymethylcytosine and ten-eleven translocation dioxygenases in head and neck carcinoma
Source: J Cancer. 2019 Aug 28;10(21):5306–14. doi: 10.7150/jca.34806 (PMC6775623; doi:10.7150/jca.34806)
Supplement: Supplementary file 1 — Supplementary figure and table. [file jcav10p5306s1.pdf]

Figure S1 Kiyoshi Misawa

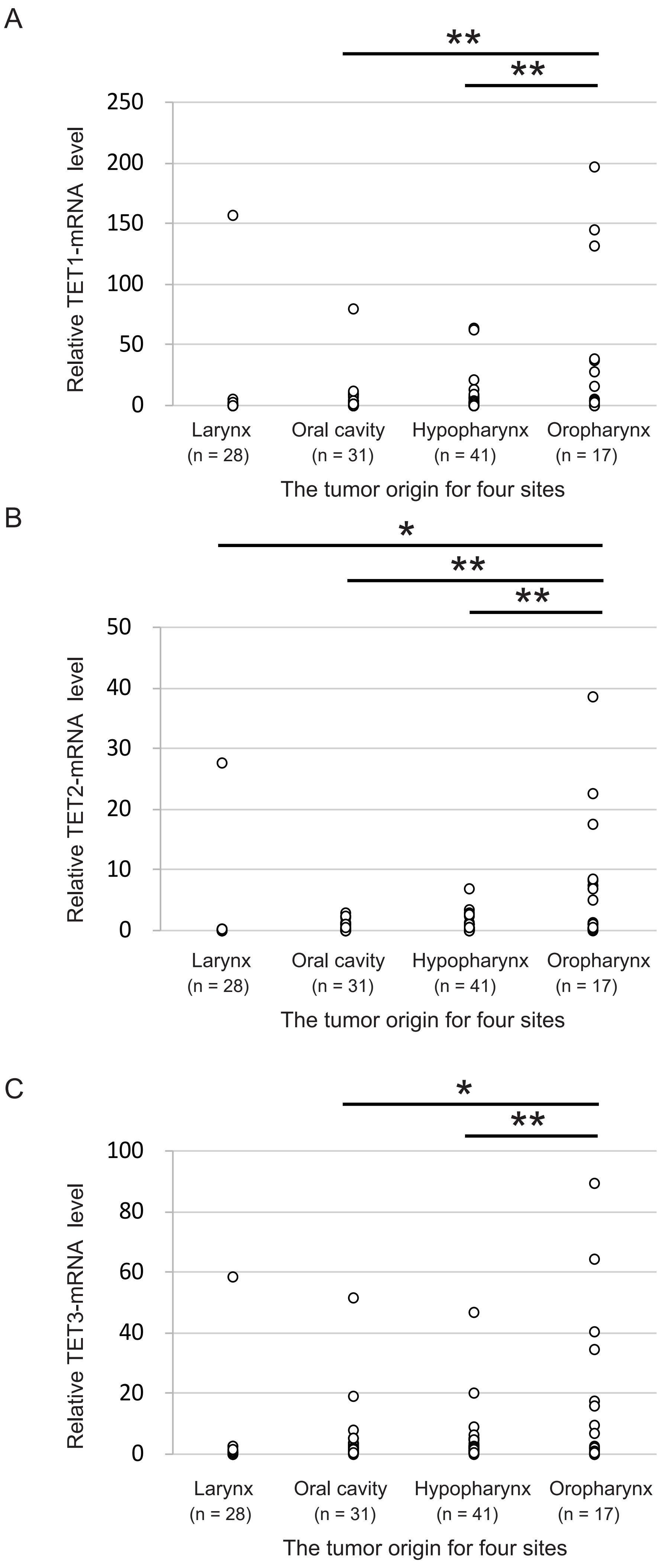

Table S1. Baseline characteristics of the HNSCC patients (n = 117)

Characteristic	No. of patients (%)
Age	
< 65	42 (35.9%)
> 65	75 (64.1%)
Sex	
Female	14 (12.0%)
Male	103 (880%)
Tumor location	
Hypopharynx	41 (35.0%)
Larynx	28 (23.9%)
Oropharynx	17 (14.5%)
Oral cavity	31 (26.5%)
Alcohol exposure	
drinker	94 (80.3%)
non drinker	23 (19.7%)
Smoking status	
smoker	98 (83.8%)
non smoker	19 (16.2%)
HPV status	
positive	14 (12.0%)
negative	103 (88.0%)
Tumor size	
T1	9 (7.7%)
T2	39 (33.3%)
T3	28 (23.9%)
T4	41 (35.0%)
Lympho-node status	
N0	46 (39.3%)
N+	71 (60.7%)
Stage	
I	7 (6.0%)
II	13 (11.1%)
III	26 (22.2%)
IV	71 (60.7%)
Recurrence events	
positive	45 (38.5%)
negative	72 (61.5%)
